# Effect of High-Current Pulsed Electron Beam on Properties of Graphene-Modified Aluminum Titanium Carbide Composites

**DOI:** 10.3390/ma15227879

**Published:** 2022-11-08

**Authors:** Ying Zhang, Guanglin Zhu, Bo Gao, Lei Wang, Zongbin Li, Liang Hu, Zeyuan Shi, Qihao Yin

**Affiliations:** 1 Key Laboratory for Ecological Metallurgy of Multimetallic Mineral (Ministry of Education), Northeastern University, Shenyang 110819, China; 2 Key Laboratory for Anisotropy and Texture of Materials (Ministry of Education), School of Materials Science and Engineering, Northeastern University, Shenyang 110819, China

**Keywords:** high-current pulsed electron beam, Al-20TiC composite, electrical conductivity, microhardness

## Abstract

High-current pulse electron beam (HCPEB) is an advanced surface modification technology developed in recent decades. This paper focuses on the effect of 0.3 wt.% graphene on the electrical conductivity and microhardness of HCPEB-treated Al-20TiC composites. The SEM results show that the titanium carbide was uniformly distributed in the aluminum matrix of the initial sample. Conversely, the graphene showed a small aggregation, and there were holes and cracks on the top surface of the sample. After HCPEB modification, the agglomeration of graphene gradually improved, and the number of surface pores reduced. The X-ray diffraction results show that after HCPEB treatment, the aluminum diffraction peak widened and shifted to a higher angle and the grain was significantly refined. Compared with the initial Al-20TiC composite samples, the conductivity of graphene-modified HCPEB-treated sample increased by 94.3%. The microhardness test results show that the microhardness of the graphene-modified HCPEB-treated sample increased by 18.4%, compared with the initial Al-20TiC composite samples. This enhancement of microhardness is attributed to the joint effects of fine grain strengthening, dispersion strengthening of the second phase, solution strengthening and dislocation strengthening. In brief, HCPEB has good application prospects for powder metallurgy in future.

## 1. Introduction

Metal matrix composites are made of iron, aluminum, magnesium, copper and other metals or alloys. Meanwhile, metal or inorganic non-metal is often added to prepare metal matrix composites with higher performance [1,2,3]. Among them, the aluminum matrix composites with high strength, small density, high impact resistance, low thermal expansion coefficient, high modulus and wear resistance have attracted the attention of an increasing number of researchers. However, they are prone to instantaneous fracture and low ductility.

Among metal matrix composites, particle-reinforced metal matrix composites have been rapidly developed in recent years. Furthermore, they can be further combined with other common material technologies for secondary processing. TiC as a ceramic particle, with their relatively cheap price, simple preparation technology, higher modulus and good toughness, can be a good additive material in the aluminum substrate. The microhardness and wear resistance of metal matrix composites can significantly increase after adding a certain content of TiC. At the same time, graphene with high-performance mechanical properties and low density is the ideal reinforcement material. Zhang [4] prepared graphene/aluminum nanocomposites using a friction stirring processing combined with a hot extrusion process, sand it was found that graphene could be uniformly dispersed in the aluminum matrix, providing a structural basis for improved mechanical properties. Bustamante [5] et al. successfully prepared graphene/aluminum matrix composites and it is found that increasing the ball milling time effectively improved the dispersion of graphene in the matrix, thus further enabling the excellent properties of graphene to be fully exploited. The microhardness of their graphene/aluminum matrix composites was increased by a factor of 138% compared to that of pure aluminum. As a result, the introduction of graphene into the aluminum matrix is expected to further improve the overall performance of aluminum matrix composites, which can meet the requirements of wear resistance, electrical conductivity, mechanical strength and hardness in specialised environments [6,7].

Traditional preparation methods for aluminum matrix composites include casting (stirring and pressure), pressureless infiltration, spray deposition, in situ reaction techniques and powder metallurgy [8,9,10]. The casting method has some problems, such as segregation, interface reaction and limited volume fraction of reinforcement. There are some problems in non-pressure infiltration, such as coarse grain size and difficult-to-control interfacial reaction. The jet deposition method is used to form fine pores and low material density. In the in situ synthesis method, the in situ synthesis system is limited and the product density is not high. The powder metallurgy method can control the degree of interfacial reaction between the two well; the selection of reinforcement is large and the powder particles can be dispersed well. In addition, graphene has a perfect, ideal structure, which determines its extraordinary properties. High-current pulsed electron beam technology can solve the above problems. As a high-density energy source, HCPBE can achieve energy deposition on the material surface in a short time, irradiating the metal surface for rapid heating and cooling [11,12,13,14,15]. According to the literature, HCPEB treatment can refine the surface microstructure of metal, induce the melting of the aluminum matrix on the surface, fill the pores of the material and greatly improve the wear resistance, hardness and other properties [16,17,18,19,20,21]. However, few research works have reported on the effects of electron beams on the electrical conductivity of a treated sample. Therefore, in this study, graphene-modified aluminum matrix composites were prepared by the powder metallurgy method. The surface modification of the composites was performed by HCPEB with the aim of improving the hardness, wear resistance and conductive properties of the composites.

## 2. Experimental Procedure

### 2.1. Preparation of Materials

Commercial powders were used in this experiment, such as aluminum powder, titanium carbide powder and graphene. The composition and particle sizes of the raw materials are shown in Table 1.

The raw materials used in this experiment were commercial Al powder (Shenyang Jiabei Trading Co, Ltd., China), TiC powder (Qinghe County Kete New Material Technology Co, Ltd., China), and graphene (Suzhou Gao Qiao New Material Technology Co, Ltd., China). The powder metallurgy process was used to prepare Al-20TiC and Al-20TiC-0.3G composite materials. The specific preparation process was as follows: To prepare for ball milling, Al powder, TiC powder, graphene (graphene is not required for Al-20TiC composites in this step) and 1 wt.% hydroxypropyl methylcellulose (HPMC) binder, as well as zirconia agate balls, were weighed to place in the ball milling tank with a ball-to-material ratio of 3:1. Finally, the above ball mill mixture was mixed in a roller ball mill for 2 h. The mixed powder was then loaded into a groove in a steel mold for cold isostatic pressing to produce a raw billet with dimensions of 50 mm × 10 mm × 5 mm, with a holding pressure of 270 MPa for 3 min during the molding process. Next, the raw embryos were sintered in a tubular resistance furnace at 590 °C with a heating rate of 9 °C/min and held for 7 h to produce Al-20TiC and Al-20TiC-0.3G composites. Before the HCPEB treatment, a metal cutting machine was used to cut the composite material into metal blocks with dimensions of 10 mm × 10 mm × 5 mm. Then, different specifications of water-resistant sandpaper (80#, 180#, 240#, 600#, 800# and 1500#) were used for grinding and, afterwards, diamond polishing paste (2.5 µm, 1 µm particle size) was used for mechanical polishing. The polished samples were sonicated with absolute ethanol for 7 min and then dried with a hair dryer in preparation for electron beam processing.

### 2.2. HCPEB Treatment

The surface modification of the materials uses a HOPE-I type HCPEB device manufactured by the Dalian University of Technology (Dalian, China). The corresponding process parameters were as follows: the pulse duration was 2.5 µs; the electronic acceleration voltage was 24 kV; the energy density was 2 J/cm^2^; the transmitting stability was >90%; the pulse interval was 10s; processing ranges: radial was 300 mm, axial was 200 mm and vacuum degree was 6.5 × 10^−3^ Pa; and the pulse times were 5, 15 and 25 times, respectively.

### 2.3. Microstructure Characterisation and Performance Analysis

The microstructures of aluminum matrix composites before and after HCPEB treatment were characterized by field emission scanning electron microscopy (TESCAN MIRA3, Tescan, Shanghai, China). X-ray diffractometer (Model XRD-7000, Shimadzu Co, Ltd., Tokyo, Japan) was used for surface phase analysis. Cu target Kα radiation was used in the experiment; graphite monochromator filter was used; the characteristic wavelength was λ = 1.5406 A step scanning; the step length was 0.02°; the acceleration voltage was 40 kV; the current was 40 mA. The scanning range was 30° to 100°, and the scanning speed was 3°/min. The wear resistance of the aluminum matrix composites was measured by the reciprocating friction test in the MFT-4000 multifunctional material surface performance tester. The scratch length was 7 mm and the loading rate was 2 N/min. The surface microhardness of the aluminum matrix composites was measured by a HVS-50Z digital Vickers hardness tester. The surface of the material was pressed by a normal diamond quadric pyramid indenter with a top angle of 136°, and the indentation depth and size was tested under the conditions of a 200 g test force for 15 s to determine the hardness of the material. The electrical conductivity of the aluminum matrix composites before and after HCPEB treatment was tested by a ST2235 resistivity tester manufactured by Shanghai Zhengyang Instrument Co, Ltd., China. The electric bridge method was used to test the conductivity of the material surface. The room temperature was 23.8 °C. The size of the test sample was 10 mm × 7 mm × 4 mm, and the average value of each sample was taken after three measurements. The measuring range of the equipment was 0.01 mΩ~11 Ω.

## 3. Results and Discussion

**Figure 1:**
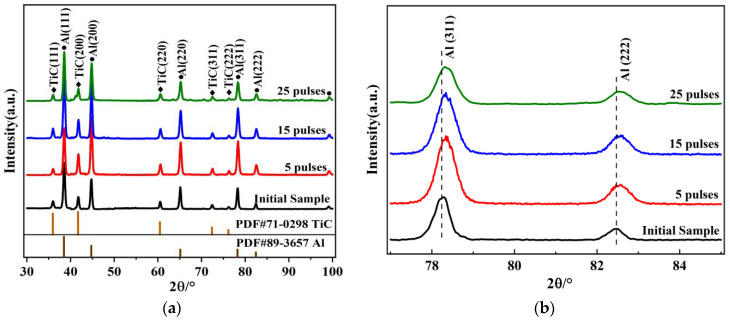
XRD patterns of Al-20TiC composites before and after HCPEB treatment: (**a**) complete XRD pattern; (**b**) local enlargement.

Figure 1 shows the XRD patterns of the Al-20TiC composite materials before and after HCPEB treatment. It was found that, compared with the initial sample, no new diffraction peak appeared after HCPEB treatment (as seen in Figure 1a) and no new phase was formed on the surface of the Al-TiC composite material. In addition, it was found that the Al diffraction peak broadens and grains were refined after HCPEB treatment, and the change became more obvious with the increase in pulse time. All the diffraction peaks shifted to a high angle (right shift), and the maximum deviation angle appeared in the XRD diffraction pattern of the five-pulse-treated sample. The phenomenon of the Al diffraction peak (311 crystal plane) broadening and shifting to a high angle was mainly due to the combined effects of grain refinement and the formation of a compressive stress state after electron beam treatment [22]. In addition, the strongest aluminum diffraction peak of the initial samples appeared on the crystal plane (111), but the strongest peak appeared on the crystal plane (200) after the five-pulse treatment, indicating that the preferred orientation of the aluminum grains changes after HCPEB treatment. Previous studies [23,24] showed that HCPEB treatment could change the crystallographic texture of the surface of metal samples, resulting in the change of the preferred orientation of crystal planes.

**Figure 2:**
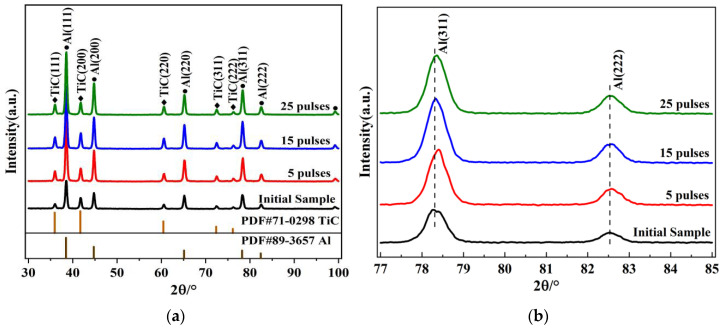
XRD patterns of Al-20TiC-0.3G composites before and after HCPEB treatment: (**a**) complete XRD pattern; (**b**) local enlargement.

Figure 2 shows the XRD patterns of the Al-20TiC-0.3G composites before and after HCPEB treatment. Compared with Figure 1, no new phase was formed after HCPEB treatment for the graphene-modified samples. It was also found that the Al diffraction peak was amplified, widened and migrated; the diffraction peak migrated first to the high angle and then to the low angle, and the maximum deviation angle appeared in five-pulse-treated sample. No carbon was detected in the XRD pattern, mainly because the amount of graphene added to the composite was too small to be identified, and its content was below the detection limit of XRD (about 1%).

**Figure 3:**
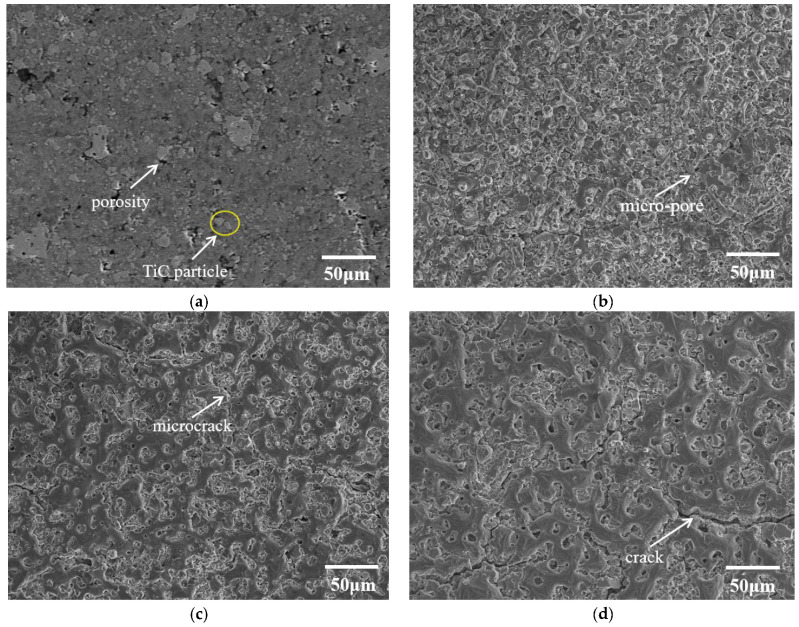
SEM images of Al-20TiC composites before and after HCPEB treatment: (**a**) initial sample; (**b**) 5-pulse-treated sample; (**c**) 15-pulse-treated sample; (**d**) 25-pulse-treated sample.

Figure 3 shows the surface microstructure of the Al-20TiC composites before and after HCPEB treatment. Figure 3a provides the microstructure of the initial Al-20TiC sample; it shows that the titanium carbide particles and aluminum matrix are coarse, with particle sizes of 20~50 μm, and the segregation phenomenon was very serious. Meantime, it can be observed that a certain amount of pore structure was distributed in the vicinity of the titanium carbide particles. Studies [25] have shown that when the sintering temperature is 590 °C, the liquid Al has a high viscosity, which leads to a relatively poor fluidity of liquid aluminum, making the material unable to complete its feeding during solidification and, finally, forming a pore structure. After HCPEB treatment, the surface morphology of Al-20TiC was changed, and it can be clearly seen that the large pores on the surface of the Al-20TiC were significantly reduced. After 15- and 25-pulses irradiation, the micropores on the surface of the material decreased. In addition, Figure 3c shows that after 15-pulse treatments, microcracks appear on the surface, which were caused by the melting of the material surface caused by the electron beam treatment. During the subsequent rapid solidification process, the volume of the surface layer shrunk by default. The subcooled matrix beneath the melting layer inhibited this shrinkage, leading to the generation of tensile stress. Figure 3d shows that the large microcracks on the surface of the 25-pulse-treated sample increased significantly, which was due to the accumulation of tensile stress on the material surface with the increase in pulse times. This stress promoted the initiation and expansion of cracks, leading to the formation of microcracks.

**Figure 4:**
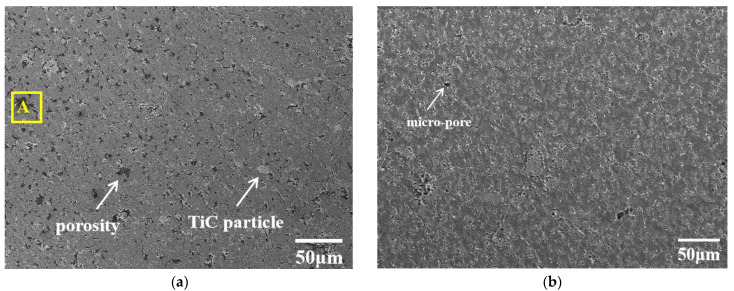

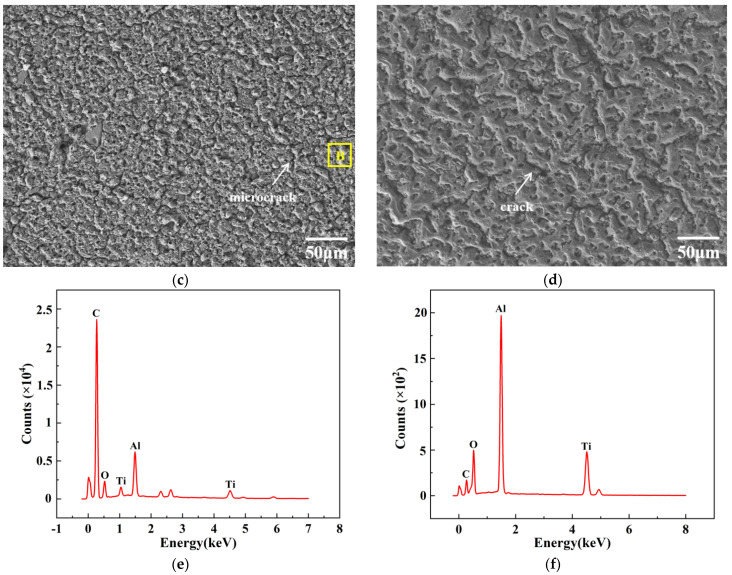
SEM images of Al-20TiC-0.3G composites before and after HCPEB treatment: (**a**) initial sample; (**b**) 5-pulse-treated sample; (**c**) 15-pulse-treated sample; (**d**) 25-pulse-treated sample; (**e**) energy spectrum of point A in original tissue; (**f**) energy spectrum of point B of 15-pulse-treated sample.

**Figure 5:**
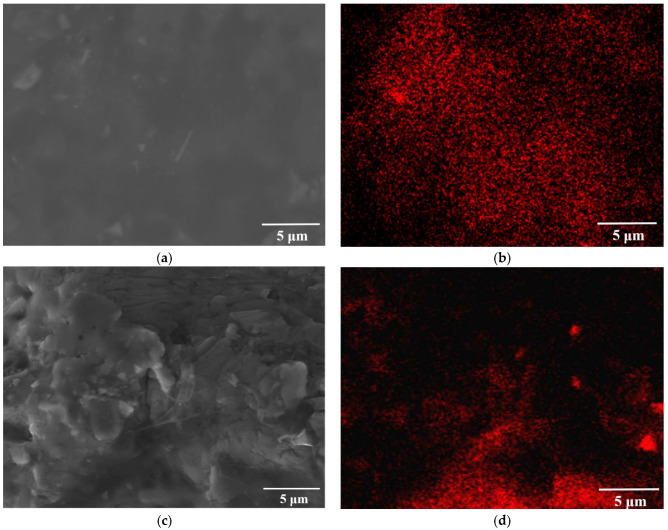
Secondary electron and backscattered electron image of Al-20TiC-0.3G composites before and after HCPEB treatment: (**a**) initial sample; (**b**) element distribution of C; (**c**) 15-pulse-treated sample; (**d**) element distribution of C.

**Figure 6:**
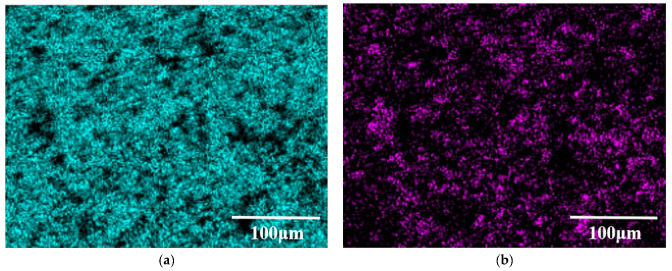

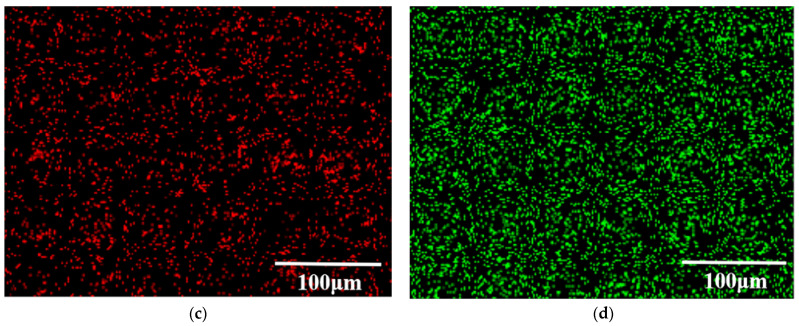
Surface element distribution of Al-20TiC-0.3G initial sample: (**a**) element distribution of Al; (**b**) element distribution of Ti; (**c**) element distribution of C; (**d**) element distribution of O.

**Figure 7:**
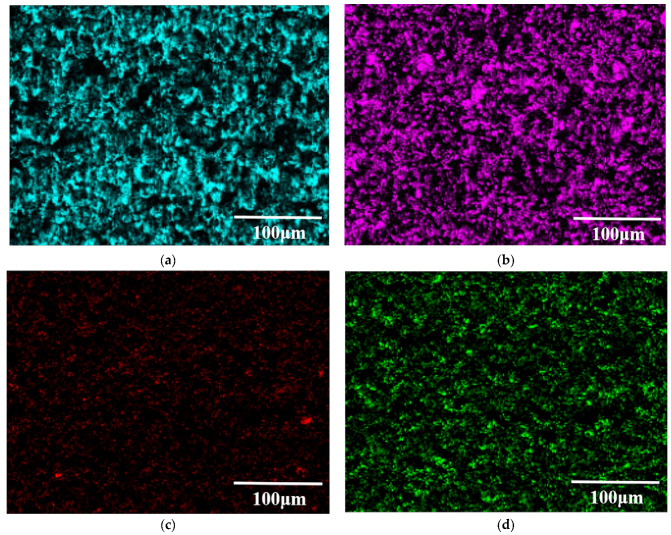
Surface element distribution of Al-20TiC-0.3G with 15-pulse-treated sample: (**a**) element distribution of Al; (**b**) element distribution of Ti; (**c**) element distribution of C; (**d**) element distribution of O.

Figure 4 shows the surface microstructure of the Al-20TiC-0.3G composite before and after HCPEB treatment. Compared with the initial surface morphology of the Al-20TiC composite in Figure 3a, it was found that after the addition of graphene, the distribution of the TiC particles in the aluminum matrix was more uniform. The material surface has no obvious metallurgical defects, and the distribution of graphene was relatively uniform, and did not show an obvious segregation phenomenon. In order to further prove the existence and dispersion of graphene in the aluminum matrix, the composite materials were observed by scanning electron microscopy at high magnification, as shown in Figure 5. Wang [26] et al. prepared graphene-reinforced pure aluminum and aluminum matrix composites, and the results showed that when the graphene content was 0.5 wt.%, graphene was uniformly dispersed in the grain boundaries of the aluminum matrix, which promoted the movement of phonons in the matrix material and improved the hardness and corrosion resistance of the composites. Therefore, the addition of graphene into the aluminum matrix composites in this study was 0.3 wt.%, graphene can change the crystal structure of the matrix material, and the stress field around graphene can interact with the dislocation stress field to hinder the dislocation movement and improve the metallurgical defects. As shown in Figure 4b–d, the large size pores on the surface of the sample were significantly reduced after HCPEB treatment, because the aluminum matrix with a low melting point in the composite was rapidly melted and filled into the pores by the rapid melting technology. The shock wave generated by the HCPEB treatment also had a certain compaction effect on the deep pores. In addition, it can be seen in Figure 4e,f that the Al, Ti and C phases exist on the sample surface before and after the HCPEB treatment. The distribution of the surface elements in Figure 4a,c is shown in Figure 6 and Figure 7, respectively. Moreover, the reactive oxygen species react with the aluminum matrix to generate alumina, which covers the surface of the material and fills into the microcracks and other defect structures brought by electron beam, effectively reducing the density of the microcracks.

Figure 5 shows the secondary electron and backscattering electron patterns of the Al-20TiC-0.3G, which shows the distribution of graphene in the Al matrix composite. As shown in Figure 5a,b, the graphene was uniformly distributed in the aluminum matrix, with thicker graphene embedded in some positions, possibly due to π–π interaction between graphene sheets. As shown in Figure 5c,d, C was locally enriched after electron beam treatment, which is because the graphene was in an unstable state after treatment.

**Figure 8:**
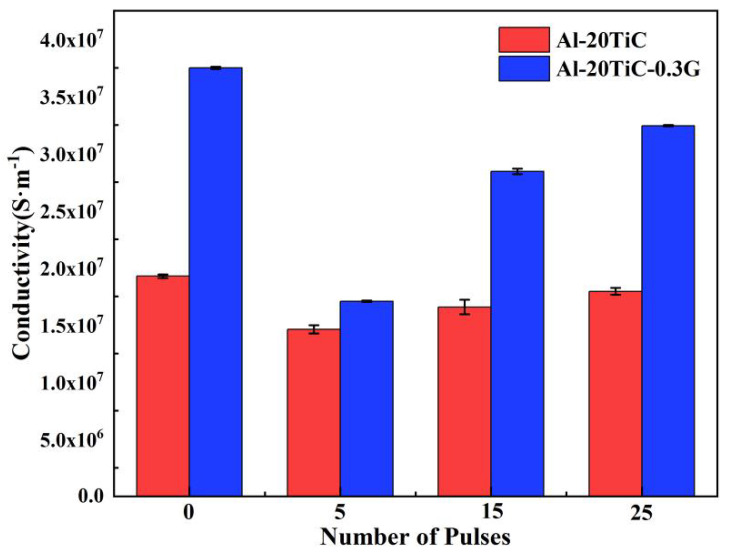
Changes in electrical conductivity of Al matrix composites before and after HCPEB.

Figure 8 shows the electrical conductivity changes of the Al matrix composites before and after HCPEB treatment. The electrical conductivity test results show that the electrical conductivity of the Al-20TiC composite was 1.93 × 10^7^ S·m^−1^; the conductivity of Al-20TiC-0.3G composites was 3.77 × 10^7^ S·m^−1^ after HCPEB treatment, which is 94.3% higher than that of the Al-20TiC composites. Studies [27] have shown that when the content of graphene is less than 0.5 wt%, there is no agglomeration phenomenon of the graphene in the composite material, and the graphene is evenly distributed in the aluminum matrix. Meanwhile, the sintering density of the composite material tends to increase, and the excellent conductivity of graphene can be fully realized. Before the content of graphene reaches 0.5 wt.%, there are basically no highly adverse factors regarding its conductivity, so the aluminum matrix composites with 0.3 wt.% graphene show an increasing trend of conductivity. After HCPEB treatment, the electrical conductivity of the Al-20TiC and Al-20TiC-0.3G composites is reduced, which is because the composite grain is refined, the grain boundary is increased, the electron scattering phenomenon is serious and some areas of C are enriched. In addition, the electrical conductivity of the Al-20TiC-0.3G composite is higher than that of Al-20TiC composite before and after HCPEB treatment because of the ultra-high electrical conductivity of graphene itself; adding to the aluminum matrix will definitely benefit the electrical conductivity of the composite.

**Figure 9:**
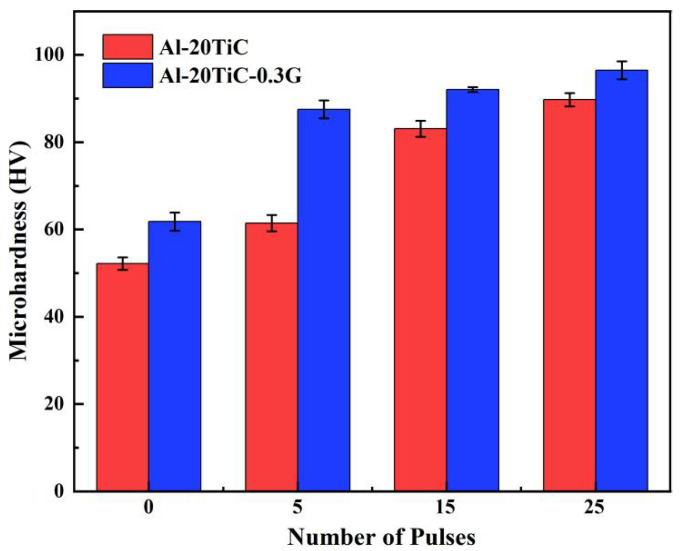
Changes in microhardness of electron beam treated aluminum matrix composite surfaces before and after the addition of graphene.

Figure 9 shows the changes in microhardness of the aluminum matrix composites (Al-20TiC and Al-20TiC-0.3G) before and after HCPEB treatment. It can be clearly seen that the surface microhardness of the two aluminum matrix composites significantly increased after HCPEB treatment. In addition, the trend of increasing microhardness became more and more obvious with the number of pulses and the addition of graphene. Before electron beam treatment, the average values of Al-20TiC and Al-20TiC-0.3G microhardness were 52.2 HV and 61.8 HV, respectively, and after 25 pulse treatments, Al-20TiC microhardness increased to 89.8 HV and 96.5 HV, increases of 72% and 56.1% compared with the initial samples, respectively. The microhardness value increased by 18.4% after the addition of graphene.

The improvement of the surface microhardness of the Al-20TiC composites by HCPEB treatment can be explained by the following aspects: First, the electron beam pulse treatment led to the refinement of coarse grains on the surface of the aluminum matrix, and a large number of fine titanium carbide particles were distributed in the aluminum matrix, which plays the role of fine grain strengthening and second phase dispersion strengthening, thus improving the microhardness of the material surface. Second, the electron beam effectively eliminated the agglomeration of graphene, and 0.3% graphene was uniformly distributed on the aluminum matrix, thus improving the hardness of the composite.

**Figure 10:**
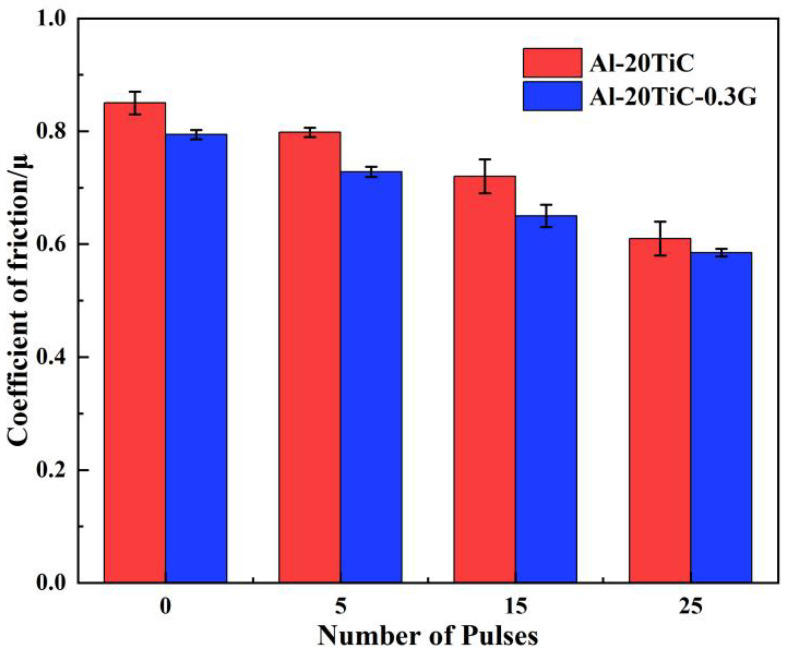
Variation of friction coefficient of Al-20TiC and Al-20TiC-0.3G composites with different number of pulses.

Figure 10 shows the changes in the friction coefficients of the Al-20TiC and Al-20TiC-0.3G composites at different pulse times. It can be seen that the friction coefficient of the surface of the Al-20TiC-0.3G composites shows an overall trend of decreasing with the increase in pulse times, and the friction coefficient of the specimen before HCPEB treatment was 0.794. The friction coefficient on the surface of the specimen reached the minimum value of 0.585 at 25 pulses: a decrease of 26.3%. In addition, it can be seen in Figure 7 that the surface friction coefficient of the Al-20TiC-0.3G composite was significantly smaller than that of Al-20TiC composite.

## 4. Conclusions

In this paper, the effects of graphene on the properties of Al-20TiC composites after HCPEB treatment were investigated and the following conclusions were drawn:

(1) The XRD results showed that no new phases were generated on the surface of the aluminum matrix composites after the electron beam treatment, and the relative intensities of the diffraction peaks changed, which may be due to the selective orientation and the change of the weave coefficients after the HCPEB treatment.
(2) The scanning electron microscopy results show that the addition of graphene makes the TiC particles more uniformly distributed in the aluminum matrix, and the composite has a good surface without obvious metallurgical defects. Meanwhile, the rapid melting and solidification effect of the electron beam makes the molten aluminum fill and reduce the pores.
(3) The conductivity test results showed that the addition of graphene to the aluminum matrix substantially improved the conductivity of the aluminum matrix by 94.3%.
(4) The microhardness test results showed that, compared with the initial Al-20TiC composite samples, the microhardness of graphene-modified HCPEB-treated sample increased by 18.4%.
(5) The results of the wear resistance test show that the friction coefficient of the sample surface reached the minimum value at 25 pulses from 0.794 in the initial sample to 0.585 in the 25-pulse-treated Al-20TiC-0.3G composites.

## Figures and Tables

**Table 1:** Composition and granularity of raw materials.

Powder	Purity/wt.%	Particle Size/μm
Al	99.9	50
TiC	99.9	10
Graphene	97.5	0.001–0.003

## Data Availability

Data will be made available upon request from corresponding author.
